# Three-Dimensional Printing of Multifunctional Composites: Fabrication, Applications, and Biodegradability Assessment

**DOI:** 10.3390/ma16247531

**Published:** 2023-12-06

**Authors:** Beata Anwajler, Anna Witek-Krowiak

**Affiliations:** 1 Department of Energy Conversion Engineering, Faculty of Mechanical and Power Engineering, Wroclaw University of Science and Technology, 27 Wybrzeze Wyspianskiego Street, 50-370 Wroclaw, Poland; 2 Department of Advanced Material Technologies, Faculty of Chemistry, Wroclaw University of Science and Technology, 27 Wybrzeze Wyspianskiego Street, 50-370 Wroclaw, Poland; anna.witek@pwr.edu.pl

**Keywords:** multifunctional composites, biodegradability composites, 3D printing, AM technology, biomass, methods assessing biodegradability, natural fillers, biofibers, biocomposites

## Abstract

Additive manufacturing, with its wide range of printable materials, and ability to minimize material usage, reduce labor costs, and minimize waste, has sparked a growing enthusiasm among researchers for the production of advanced multifunctional composites. This review evaluates recent reports on polymer composites used in 3D printing, and their printing techniques, with special emphasis on composites containing different types of additives (inorganic and biomass-derived) that support the structure of the prints. Possible applications for additive 3D printing have also been identified. The biodegradation potential of polymeric biocomposites was analyzed and possible pathways for testing in different environments (aqueous, soil, and compost) were identified, including different methods for evaluating the degree of degradation of samples. Guidelines for future research to ensure environmental safety were also identified.

## 1. Introduction

Over the past few decades, our environment has witnessed a significant accumulation of plastic waste, mainly due to human economic activities. Plastic pollution negatively affects the ecosystem and global warming, so our society urgently needs solutions to counteract these effects. Therefore, the closed-loop economy principle is fundamental to reducing the amount of non-biodegradable petroleum-based waste [1]. This article considers a production process for manufacturing products based on 3D printing. This interest stems from the great possibilities offered by this technology, i.e., the wide range of materials used, including recycled and waste materials that can be printed, and the short time and low cost of printing itself (e.g., FDM 3D printing) [2]. However, the main focus is developing new materials that could replace conventional petroleum-based polymers, offering specific properties tailored to different applications, while remaining sustainable, technologically, and economically viable [3].

In recent years, additive manufacturing (AM) technology has attracted increasing attention, and its intensive development has led to a shift from the rapid prototyping of parts to the production of commercial components, becoming an aspect of the transformation of Industry 4.0 [4,5]. Additive manufacturing technology has come a long way since its inception when Chuck Hull, co-founder of 3D Systems, developed the first 3D printer in 1983 [6]. In the years since, interest in the technology has grown and become more affordable and accessible. It is an innovative manufacturing process that builds three-dimensional parts directly from digital model files by combining materials layer by layer [7]. AM is considered one of the key technologies for transforming traditional manufacturing into smart manufacturing, which aims to use advanced technologies to make products. It is capable of producing parts from micro to macro scale, with precision and accuracy tied to specific printing techniques and parameters [8]. Three-dimensional printing offers excellent potential for reducing environmental impact by enabling the production of complex and high-quality parts from various possible materials with minimal waste. The construction industry in China has successfully printed a group of houses in less than a day. In addition, the technology has been recognized as an effective solution for treating tissue defects in the biomedical field, as various medical implants and scaffolds can be produced using 3D printing [8]. 

One of the main advantages of AM is its ability to combine with composite materials, the reinforcement phase in polymeric, ceramic, or metallic matrices. As a result, the mechanical and physical properties of the materials can be tailored to specific applications. These attractive factors of 3D printing are used to produce complex parts in various industries such as architecture [9], biomedical [10], aerospace [11,12], and food [13]. The main disadvantages of 3D printing include limited material usage, post-processing requirements, limited print size, low printing speeds, limited part size, and durability. These issues can be mitigated by combining reinforcement and matrix composites to achieve functional properties that are impossible with single components. Available properties such as thermal conductivity, electrical conductivity, actuation, sensing, and self-healing capabilities are achieved by incorporating additives or adjusting component design [14]. The synergistic effect of the resulting structure drives traditional materials toward advanced functional materials, thereby enriching material properties. Multi-material and multi-scale structures offer a potential way to optimize overall component properties [14,15].

This review aims to provide valuable insight into the prospects of AM technology by discussing recent advances in the 3D printing of polymeric materials and their composites, as well as a method for evaluating the biodegradability of the produced composites.

## 2. Multi-Material 3D-Printed Polymer Composites

Based on the International Organization for Standardization (ISO) and the American Society for Testing and Materials (ASTM) standard 52.900:201 [16], 3D printing techniques are divided into seven categories consisting of binder injection (BJ), directed energy deposition (DED), material extrusion (ME), material sputtering (MJ), powder bed fusion (PBF), sheet lamination (SL), and vat photopolymerization (VP) [1,2]. Each AM method has its specific applications based on its advantages. For example, selective powder bed fusion techniques are best suited for producing intricate and accurate parts. On the other hand, if we care about the highest deposition rate due to the raw material, filament AM methods, commonly used for large-scale production of components, will be the most suitable [3]. The multifunctionality of composites [1,2,3] combines different properties in a single material or structure. This allows them to perform two or more functions, such as optical, magnetic, electronic, thermal, or structural, making the resulting composites suitable for the applications in which they will be used. This has the advantage of achieving autonomy, adaptability, self-sufficiency, and weight reduction. Combining two or more properties in a single material is desirable in all engineering fields, from robotics to life sciences, to serve as embedded structural health monitoring from cradle to death or as structural energy storage. Accordingly, the interest of researchers in multifunctional and multi-material polymer-based composites continues to grow, especially in nanoscale materials, biomimetics, structural energy composites, etc. [2,4,5]. The main advantage of 3D printing in this context is the ability to deposit the required materials precisely in specific areas of the structure, reducing manufacturing costs and time of the target composites. This gives rise to different materials and methods for creating multi-material systems. Based on the available literature, we can distinguish two methods for manufacturing multifunctional composites. These include 3D printing of composites and 3D printing of multiple materials. The difference between the two lies in how the composition of the printed composites is combined. The first method combines materials before printing, while the second uses 3D printing techniques to combine materials [2]. It primarily uses polymers and other functional inorganic or organic materials to combine multiple materials into a functional whole. Several methods are used to obtain multilayer materials, including multiwire/multilayer printing, core/coaxial rugation, and embedded printing [2,4].

### Three-Dimensional Printing of Composites

In 3D printing of composites, a matrix of polymers is combined with specific additives to produce materials with improved structural or functional properties. The resulting properties cannot be achieved by using any of the components alone [2,6,7]. Functional fillers consisting of inorganic particles or fibers are used as additional materials. A characteristic feature of this method is that the composites are prepared beforehand and then used in the 3D printing process in the same way as the pure material. As mentioned earlier, adding foreign filler particles to the polymer matrix enables high printing accuracy with improved mechanical and functional properties. Such materials are known as polymer matrix composites (MPCs). Most of the current research is focused on developing new composite materials with reinforced particles, fibers, nanomaterials, and ceramics and their use in additive manufacturing processes [2,7,8,14]. Examples of polymer composites reinforced with functional additives are listed in Table 1. The main objective of scientific research is to improve the printability of the material matrix, to mechanically strengthen the material matrix, to impart new properties to the composite (e.g., thermal, electrical, and magnetic properties), or to build a porous structure as a final element [2].

Research to improve the printability of the material matrix has mainly focused on using various types of nano/microparticles, including nanosilica, nanoclay, and micro-NaCl, as functional fillers. It has been shown that the physical and chemical interactions between the polymer matrix and the filler that occur during fabrication provide an opportunity to improve the viscosity of the material system [2].

As pure, homogeneous materials, polymeric materials typically have limited mechanical properties, limiting their potential applications. In contrast, combining different materials to achieve desired mechanical properties has recently become a promising method to overcome this problem. The literature shows many impressive results in developing new particle and fiber-reinforced materials. The most commonly mentioned nano/microparticles and fibers include nanosilica, nanoclay, aluminum/aluminum oxide (Al_2_O_3_), and C/SiC fibers [2,68].

Depending on the range of functionality, multifunctional materials can be divided into homogeneous and locally functionalized categories. In homogeneous 3D-printed composites, the conformal property is uniformly distributed throughout the printed part. Conversely, in locally functionalized 3D-printed composites, the desired functionality is restricted to a specific area of the structure. An overview of multifunctional 3D-printed materials is provided in Table 2. 

Adding a functional component to a polymer matrix material is now a promising solution. There is a lot of emphasis in the literature on the production of multifunctional composites, i.e., those in which the matrix material has complex functions, i.e., conductivity, magnetism, and reactivity to the environment (e.g., heat, solvent). Conductivity is essential for flexible and wearable electronics. Conductive organic/inorganic additives such as carbon nanotubes (CNTs), graphene, and polypyrrole are mainly used for this purpose. These 3D-printed conductive polymer composites have shown great potential in electronics [52,74,75,92,96]. 

Various carbon nanomaterials, such as carbon nanotubes (1D) and graphene (2D), have long been used as secondary phases to produce homogeneous, conductive composites (electrical properties) [78]. Carbon nanotubes (CNTs) are cited in reviews [50,57,75,79,125,126,127,128,129,130] as one of the most promising candidates in 3D printing for modifying inks or filaments to develop multifunctional structures. A common goal of all research on CNTs has been to successfully incorporate the conductive phase into the polymer (create a continuous conductive network) without exceeding the viscosity limit above which the ink or filament cannot be printed. In addition to CNTs, graphene [55,56,57,84,131,132,133,134] and graphene oxide (GO) [44] have also been used to prepare nanomodified inks for 3D printing.

According to the literature, porous structures can be freely obtained directly through the 3D printing process [135,136]; however, the pores constructed in this way are usually macroscopic, which limits the overall porosity. It is possible to 3D print composites filled with removable particles/components and then remove them. This is a novel way to build micro/nanoporous structures with high porosity. The most commonly cited example in the literature is the water-soluble salt NaCl. It is an ideal additive for non-aqueous systems, including thermosetting/thermoplastic polymers, photocurable resins, silicone rubber, and other polymers [2,137,138]. Various thermosetting polymers, including PCL, poly(glycerol sebacate) (PGS), PU, and epoxy resins filled with NaCl particles, were printed and then immersed in distilled water to obtain porous structures. CuSO_4_ salt has also been used to print porous polylactide-co-glycolide (PLGA) scaffolds. CuSO_4_ salt has a similar high solubility in water as NaCl salt. In addition to dissolution, materials removed using other methods, such as acid etching and pyrolysis or acid etching and drying, can be used to build porous structures. In this way, large porous structures of high complexity can be printed. These 3D-printed porous structures have played an essential role in various fields such as smart structures, flexible electronics, and tissue engineering [139].

However, the main focus is on developing new materials, primarily biodegradable materials, that could replace conventional petroleum-based polymers. At the same time, new materials should offer specific properties tailored to different applications while remaining sustainable and technologically and economically viable [3]. The remainder of this article focuses on another part of composites in AM technology (Runcorn, UK), namely partially and biodegradable composites, and presents and compares methods for evaluating the biodegradability of the materials produced.

## 3. Biodegradable Polymers in 3D Printing

**Figure 1:**
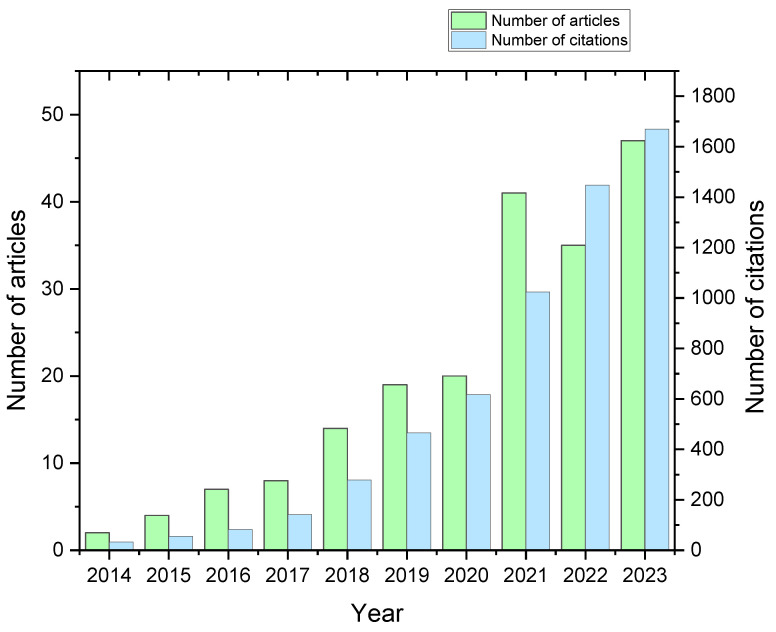
Number of publications from 2014–2023 containing selected methodology keywords (biodegradation AND (3D print) AND ((polylactic acid) OR PLA) OR (polyhydroxyalkanoate OR PHA) OR ((polybutylene succinate) OR PBS) OR ((poly lactic-co-glycolic acid) OR PLGA) OR ((polybutylene adipate terephthalate) OR PBAT) OR (polycaprolactone OR PCL) OR starch).

**Figure 2:**
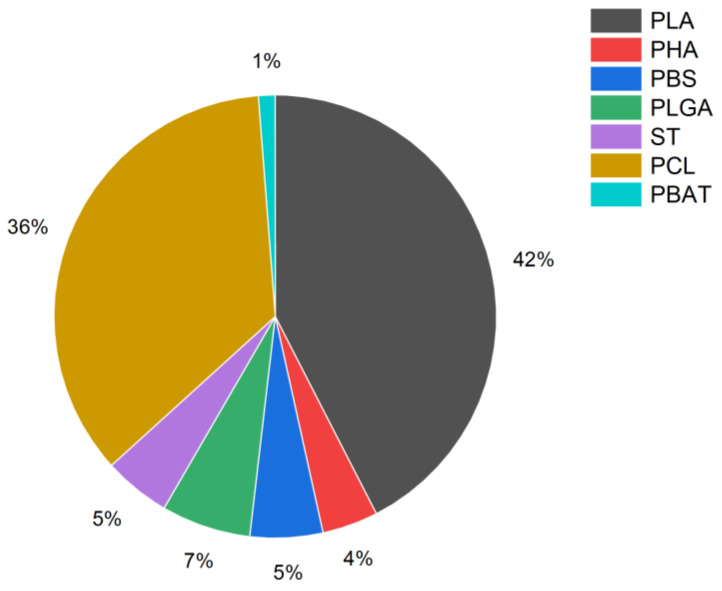
Share of specific polymers in search results selected methodology keywords (biodegradation AND (3D print) AND ((polylactic acid) OR PLA) OR (polyhydroxyalkanoate OR PHA) OR ((polybutylene succinate) OR PBS) OR ((poly lactic-co-glycolic acid) OR PLGA) OR ((polybutylene adipate terephthalate) OR PBAT) OR (polycaprolactone OR PCL) OR starch).

An analysis of published papers by researchers in the field of biodegradable materials and 3D printing technology was conducted. As a result, it was found that interest in the topic has been very evident over the last six years (Figure 1), and it is also noteworthy that the number of citations has increased more dramatically than the number of publications. In addition, PLA (more than 40%) and PCL (35.5%) dominate among the polymers studied, with other polymers accounting for a total of 25% of the cases analyzed (Figure 2).

Polylactic acid (PLA) is the most commonly used raw material in the FDM 3D printing process due to its biodegradability and environmentally friendly properties, but the use of pure PLA polymer in the FDM approach is limited due to its disadvantages such as mechanical weakness, dissolution rate in water, etc. [4]. Lactic acid can be synthesized with high efficiency from the microbial fermentation of sugars. Sugars can be obtained from sustainable or renewable plant materials. Because it can be made from renewable carbon and is biodegradable, PLA has tremendous value because other high-performance plastics, such as polyethylene and polypropylene, are not biodegradable and are made from ethylene and propylene derived from fossil fuels. Although PLA is biodegradable, it is not renewable as it emits ~1.3 kg CO_2_ equivalent/kg of synthesized plastic. Therefore, it is suggested that preparing PLA composites with appropriate additives is a feasible method to improve the properties of 3D-printed PLA parts obtained via the FDM approach [140]. 

Plastic products are primarily manufactured using injection, blow, or compression molding methods in a controlled, high-performance industrial environment. In addition, composites can be made by reinforcing plastics with fillers that include both metals and plant-based substances, including many organic compounds, which can improve the properties and surface appearance, reduce the cost, or increase the durability of composites. Three-dimensional printing has ushered in a new era in composite manufacturing, which is traditionally the domain of compression, extrusion, and injection molding. The use of biomass/lignin residues from the food, pulp and paper, forestry, and agricultural industries in biocomposites increases the efficiency of a circular economy [4,14,141]. Incorporating plant-based materials into a plastic matrix increases the use of low-cost and renewable resources and reduces the amount of plastic in the composite [4]. Using biodegradable plastics such as polylactic acid (PLA) composites can address the urgent need to replace non-biodegradable plastic composites with more environmentally friendly materials. The development of integrated biorefinery technologies has begun to expand the product portfolio of biomass utilization technologies [4,14]. The use of biomass resources in composite applications has greater flexibility than their use in the synthesis of organic compounds for the production of fuels or chemicals, which require high selectivity, high yield, and easy recovery to make the process economical [4]. Short and long lignocellulosic fibers, micro- or nanocrystalline cellulose, hemicellulose, starch, and lignin have been reinforced in thermoplastics using traditional molding methods, and composites are used commercially [9,141]. The production of biocomposites via additive manufacturing processes is expected to result in tremendous commercial growth and a great deal of scientific research has recently been conducted in this new field of advanced manufacturing [4]. Blends of biodegradable and non-biodegradable polymers are excellent raw materials for printing, providing products with improved functionality. To improve the performance of polymers and extend their functionality, additives are introduced into polymer matrices to develop composites with advanced properties compared with pure polymers [9,141,142]. For the production of biofilms, biomass resources need to be coated, sized, and treated to enable printing and ensure optimal printing properties. There are many studies in the literature where PLA filaments filled with biomass resources were printed at a nozzle temperature of 175–230 °C, a bed temperature of 25–70 °C, a layer height of 0.1–0.3 mm, and a speed of 12–75 mm/s. Most of the R&D work focusing on the effect of filler and compatibilizer on material strength is printed at 100% fill [14]. Examples of polymer/natural fiber combinations are shown in Table 3.

### 3.1. Methods for Evaluating the Biodegradability of Composites Produced Using 3D Printing Technologies

Degradation of polymer composites can occur under abiotic factors such as light, temperature, humidity, and chemical treatment. Biodegradation is a series of complex transformations that materials undergo in the presence of microorganisms and their metabolites. Compounds (enzymes and acids) produced by microorganisms aid the degradation process under environmental factors (temperature, oxygen, humidity, sunlight, etc.). The biodegradation rate is strongly dependent on the composite structure in question; monolithic blocks will biodegrade more slowly, and a highly porous polymeric structure will behave differently if the accessibility of the surface is much greater. 

**Figure 3:**
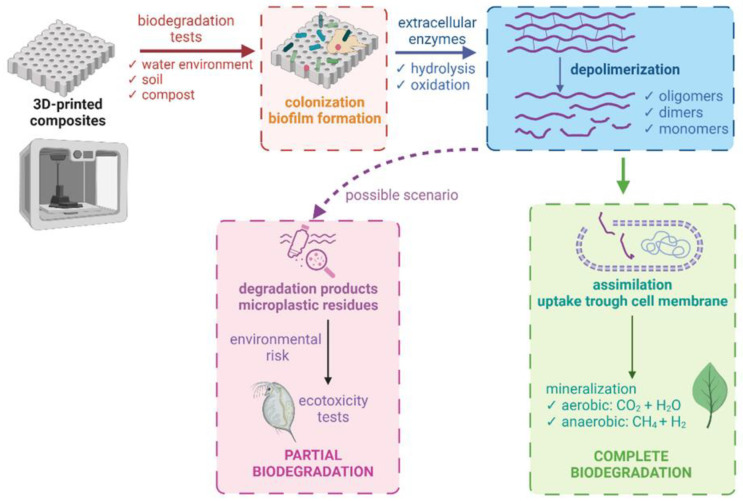
Biodegradation mechanisms (created with Biorender.com). Based on [5].

To determine whether a composite is biodegradable, appropriate biodegradation tests are required. The choice of specific tests depends on the type of composite, its application, the expected environment in which it will be placed, and industry standards and regulations. Biodegradation tests are performed under natural conditions (aqueous or soil environment) or under controlled conditions (composting or anaerobic digestion). The choice of environment is critical because each environment has different physicochemical conditions and is inhabited by different microflora. It has been shown that the same polymeric material can biodegrade at completely different rates under other conditions [190]. Microorganisms colonize the surface of polymer prints and cause the materials to degrade into shorter chains of oligomers and monomers. Polymers change their physicochemical properties as their molecular weight decreases. The overall biodegradation of materials also includes the assimilation and mineralization of molecules that are the product of decomposition by microorganisms (Figure 3). Thus, depending on the availability of oxygen, water, CO_2_, methane, and inorganic salts, the final products are different from partial degradation, which results in persistent microplastics [190]. This situation requires control of the resulting degradation products, as their presence in the environment can cause significant damage to ecosystems.

Products defined as biodegradable within 6 months will biodegrade more than 90% of their weight, while compostable products should decompose 90% in 3 months 4 [4]. However, the laboratory conditions under which biodegradation tests are conducted differ significantly from real-world conditions; the process parameters of laboratory conditions are predictable and selected to decompose materials relatively quickly. The introduction of the same material into the environment may significantly increase the biodegradation time due to the nature of the environment and its conditions.

**Figure 4:**
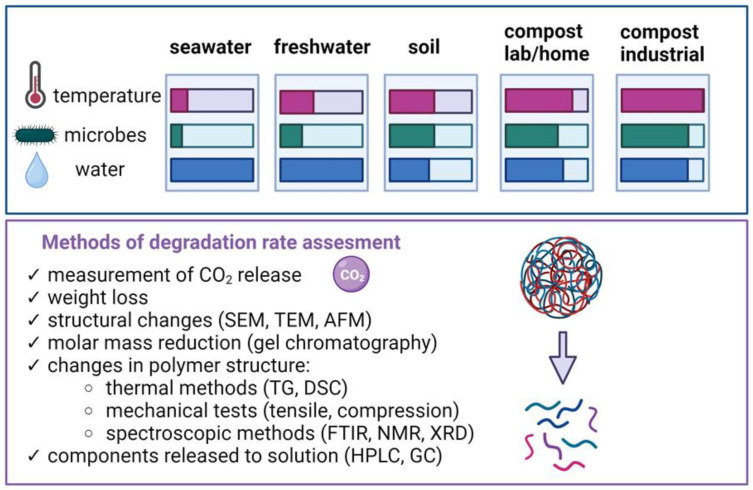
Various environments and methods to evaluate biodegradability (created with Biorender.com). Based on [5].

The natural environments in which biodegradation can occur vary widely, mainly in terms of temperature, water content, and the number of microorganisms capable of degradation (Figure 4). In most cases, we carry out processes under controlled laboratory conditions, where we greatly accelerate the possibility of biodegradation by ensuring the best process conditions. A number of different methods can be used to assess the degree of biodegradation. These methods include measuring carbon dioxide release during material mineralization, monitoring weight loss, examining surface changes (through visual or microscopic observations), and analyzing changes in composite structures. Evaluating changes in material structure involves analyzing changes in the molar mass of the polymer, using thermal techniques such as TG and DSC, evaluating mechanical properties, and using spectroscopic methods. For degradation in aqueous environments, the evaluation extends to the analysis of components released into solution from the sampled materials.

### 3.2. Biodegradation of Composites in Aquatic Environments

The degradation of composite materials can occur in the natural environment (surface water) or in an environment that mimics natural conditions (see Table 4, which shows the results of laboratory conditions that simulate fresh and saltwater and tests that mimic human body conditions for biomaterials).

#### 3.2.1. Biodegradation in Freshwater and Seawater

A large volume of plastics enters surface waters and oceans, making these environments important recipients and sites of potential biodegradation of these materials. Aquatic environments contain relatively few microorganisms compared with other environments. Aquatic environments also have a lower temperature, which means that materials collected in water will degrade much more slowly than in other habitats. There are several standards for biodegradation in aquatic environments. For example, ISO 18830:2016 [191] and ISO 19679:2020 [192] deal with biodegradation measurements under controlled conditions of seawater and sediment, with the former measuring oxygen uptake and the latter measuring the amount of CO_2_ released [193]. 

Some materials begin to degrade very rapidly in an aquatic environment. Of note is the PCL/wool composite, which begins to degrade within the first few days of entering the seawater environment. Higher wool content in the composite results in higher measured biological oxygen demand values. The study was extended to 5 months of seawater testing, during which changes in the prints (dark spots) were visually observed. The changes depended on the amount of wool used and the thickness of its fibers, which may be due to the lower amount of cuticle in thicker wool, which is more susceptible to degradation [194]. The seawater tests have been extended to include biodegradation tests in a compost environment (for filaments). The biodegradation of pure PCL in a compost environment is negligible, typically less than 1%. The presence of natural additives (undyed wool fabric waste) accelerates biodegradation up to 10 times, resulting in more than 10% degradation in 3 months, depending on the size of wool fibers of two different diameters [194]. Studies indicate that the size of biomass immobilized in polymer matrices is one of the key parameters responsible for biodegradability.

#### 3.2.2. Degradation Tests in Buffer Solutions for Medical Applications

Specific polymers are degraded in aqueous environments that mimic their future applications, such as medical applications. Such biomaterials should be safe for organisms, degrade at a certain rate, and yield non-toxic and non-inflammatory products. In vitro, room temperature, and accelerated (aging at elevated temperatures) methods estimate their biodegradation susceptibility. Materials used as implants can be tested in environments that mimic physiological environments according to ISO 10993-13:2010 [195]. Evaluation of the biodegradability of PBAT/chitosan blends confirmed the possibility of using replicas of this composite as a biodegradable cardiac occluder device [196]. Degradation of PLA scaffolds in a PBS buffer environment showed that the three-dimensional structure of the print is essential for the degradation rate. Prints with random porosity degraded the fastest, followed by cubic and gyroid [197]. It is also worth monitoring other parameters than just the change in mass of the prints, which can confirm the existence of specific degradation mechanisms of biomaterials. Interesting results have been obtained by researchers who have tested molecular weight changes during degradation. Numerous studies show that the mechanical strength of printed structures is also an important parameter, especially for tissue engineering applications. Degradation of PCL prints reinforced with natural fibers showed a significant effect of fibers on mechanical parameters (tensile and elasticity). The presence of biomass causes a faster degradation of the constructs due to the degradation of biological material. However, the values of tensile strength and modulus of elasticity are still higher than those of unreinforced PCL for up to 2 weeks [198].

Accelerated degradation at elevated temperatures resulted in a significant decrease in molecular weight without significant loss of bulk. This was explained by water diffusion into the interior of the polymer and gradual hydrolysis preceding chain degradation [199]. The accelerated degradation in an aqueous environment indicates the importance of temperature; at 50 °C, the degradation of PLA/PHA prints took much longer than at 70 °C, resulting in larger fragments. The printing direction also seems to have a significant effect; samples printed in the horizontal direction eroded, causing cracks, while samples printed in the vertical direction disintegrated completely. The presence of PHA in the PLA/PHA blend leads to a decrease in deformation during hydrolytic degradation [200].

**Table 4:** Biodegradation of 3D-printed polymeric materials in aqueous environments.

Printed Material	AM Technique	T (°C)	Time (Days)	Solution Type /Test Type	Indicators	Biodegradation Level	References
PLA, PHB, PLA/PHB	FDM	25	50	Freshwater aerobic environment, thermophilic microorganisms	CO_2_ release	PLA 8.7%, PHB 73.3%, and PLA50/PHB50 32.3%	Choe et al. [201]
PCL + wool	FDM	20	5	Marine water collected from Eastern Beach, Geelong, Australia	BS EN 1899-2:1998 [202], CO_2_ release	n.a.	Haque et al. [194]
PCL + wool	FDM	25	5 months	Domestic saltwater fish tank as an established ecosystem	Weight loss	n.a.	Haque et al. [194]
PBAT + HAp	FDM	37	30	Tris-buffer	Weight loss	6.21 for 3% HAP	Acharya et al. [203]
PBAT/Chitosan	FDM	37	168	ISO 13781:2017 [204] Sorensen buffer solution (0.2 M, pH 7.4)	Weight loss, change in molecular weight	14.17%	Wang et al. [196]
PLA	FDM	n.a.	21	PBS + 5% CO_2_	Weight loss, morphology changes (scan)	n.a.	Karimipour-Fard et al. [197]
PLA/PHB	FDM	37	195	Saline, PBS, and Hank’s solution	Solutions absorption, microscopic observation, mechanical compressive tests	n.a.	Balogová et al. [205]
PLGA	FDM	37 47	56 28	ISO 13781:2017 [204] PBS	Visual changes, weight loss, thermal properties, molecular weight change, mechanical properties	56 days at 37 °C: 2.12% mass lost, molecular weight decrease 39.5%; accelerated degradation: 4.38% mass lost, molecular weight decrease 92.4%	Ghosh Dastidar et al. [199]
PLGA/HA/CNT	FDM	37	28	PBS	Weight loss	n.a.	Kaya et al. [135]
PLC + fiber yarn	FDM	37	70	Cell culture medium, Roswell Park Memorial Institute (RPMI) 1640	Weight loss, visual changes (SEM), mechanical properties	Degradation rate 20 times higher for biomass-reinforced samples	Hedayati et al. [198]

n.a.—not applicable.

### 3.3. Biodegradation of Composites in Soil Environments 

Soil is a diverse type of environment that varies in granularity, porosity, water-holding capacity, aeration, pH, and composition of different fractions (sand, silt, and clay) [201]. An important parameter is temperature, which depends on the season and climatic conditions. Soil is home to various microorganisms, such as bacteria and fungi, which significantly impact the degradation of materials introduced into the environment. Standard methods for testing the biodegradation of plastics in soil are implemented by burying the materials in the soil at the appropriate temperature and humidity to ensure microbial activity and monitoring the release of carbon dioxide corresponding to the decomposition of the material. Methods involving mass loss and/or evaluation of properties of decomposed samples, such as morphology, structure, and surface analysis, and mechanical properties, are also used (Table 5). The effects of degradation residues on living organisms are also analyzed using ecotoxicity tests.

PLA-based composites enriched with TPS and plant biomass (Astragalus residues) showed significant weight loss (21.4%) after more than 4 months. The authors performed additional mechanical property measurements at this time, confirming the prints’ flexural strength reduction. Thermal analysis of the degraded samples revealed interesting results. The thermal stability of the composites improved, which may indicate the rapid degradation of starch and fibers in the soil, increasing the number of PLA crystalline domains in the composite [206]. Hydrophilic additives that can absorb water improve the biodegradability of PLA. The addition of thermoplastic starch and wood resulted in higher biodegradation efficiency. The activity of microorganisms initiates surface changes and allows access to the inner areas of the print, which promotes swelling and makes more space available in the composites. It has also been observed that the degree of filling of the material supports accelerated degradation [207]. Similar observations have been reported for PLA by adding rice hulls [208]. The compression pattern can influence the degree of biodegradation of polymeric materials, as demonstrated for PLA/PHA acoustic absorbers with added wood fiber. Honeycomb shapes have been shown to degrade more slowly than systems with a denser (rectilinear) structure, perhaps through better moisture uptake [209].

Blends of biodegradable and non-biodegradable polymers are excellent raw materials for 3D printing, giving products better functionality. The presence of a biodegradable polymer in the blend does not guarantee good degradation of the prototype, so it is always necessary to test these properties under real conditions. The presence of non-biodegradable polymers (HDPE and PP) in blends with biodegradable polymers causes a significant reduction in degradation, probably as a result of covering the surface of the prints with a non-biodegradable layer that resists bond cleavage, making enzymatic hydrolysis of the whole material more difficult [201]. Adding non-biodegradable polymers to PLA can improve the mechanical strength of prints. A blend of PLA and PP at the lowest possible level (7.5%) with the addition of a compatibilizer (PE-g-MAH) was designed. A full print optimization was performed using table temperature, nozzle temperature, and biodegradation time as independent variables. The system’s response was the mechanical tensile strength and weight change in the prints. Printing temperature was a statistically significant parameter with an optimum printing temperature of 171 °C. High biodegradation resistance of the proposed compound was observed [210]. 

**Table 5:** Biodegradation of 3D-printed polymeric materials in soil.

Printed Material	AM Technique	T (°C)	Time	Humidity (%)	Soil Type	Indicators	Biodegradation Level (%)	References
PLA/PHA-wood fiber	FDM	30	30 days	80	Coco peat, red scorched soil, fine sand, charcoal, and microorganisms	Weight loss	2.47–3.85%	Sekar et al. [209]
PLA/PHA-wood fiber	FDM	30	28 days	80	Coco peat, red burnt soil, fine sand, charcoal, and microbes	Weight loss	2.45%	Sekar et al. [209]
PLA-TPS-wood	FDM	30	4 months	85	Forest soil	Weight loss, thermal properties, FTIR	PLA 0.5% PLA-TPS 1–18%	Lee et al. [207]
PLA-TPS-ARP	FDM	Room	180 days	17–21.5%	n.a.	Weight loss, surface changes, mechanical properties, thermal stability, and thermal dynamic mechanic testing	21.40%	Ni et al. [206]
PLA/PP	FDM	n.a.	45 days	n.a.	n.a.	Mechanical parameters (tensile strength)	n.a.	Harris et al. [210]
PLA/rice husk	FDM	21–25	90 days	30%	n.a.	Weight loss	Weight loss up to 40%	Tsou et al. [208]

n.a.—not applicable.

### 3.4. Biodegradation in a Composter

Composting is an important alternative to landfills as an option for decomposing microorganism-sensitive materials. It can be implemented in backyard, laboratory, or industrial settings. Composting is influenced by several factors, such as temperature, humidity, pH, feedstock composition (C/N ratio), and microbial content and diversity. Large-scale composting is much more efficient and can operate under thermophilic conditions, up to 70 °C, with higher humidity and oxygen availability. Compost is a high microbial environment. The content of the bacterial population in compost can reach 109 CFU/g [5,211]. Composting can be carried out both on a small scale and under industrial conditions, but in the latter case, the most common response to biodegradation is the visual evaluation of the prints (Table 6).

As a representative of polyesters, PLA is degraded by chemical hydrolysis, which favors the degradation of this polymer in high-humidity environments. Biodegradation of PLA in a composting environment where temperature and humidity are at a high level shortens the biodegradation time compared with, for example, decomposition in soil [136]. For the PLA/PHB blend, better biodegradation results were obtained using lab-scale composting than for printing from pure PLA. Enzymatic degradation of polyesters can be realized by the action of microbial enzymes and hydrolysis, with the presence of polyhydroxy acids of microbial origin assisting the degradation process. Prints with the potential application of cosmetic packaging showed better degradability because they contain additional cosmetic residues (paraffin), an additional carbon source for microbes, and residual water, which accelerates PLA degradation [212]. PLA and PHB polymer impressions can exhibit very different biodegradability. PHB shows relatively rapid mineralization (84.6%) compared with the PLA50/PHB50 composite (biodegradation of 85%) in composting tests, indicating that PHB is more susceptible to the microbial enzymes of the compost. In the same test, the degradation of PLA prints yielded a surprisingly low result (21.7%); the extrusion and printing process may affect structural changes within this polymer [201]. PBAT, as a representative of polyesters containing an aromatic group, has a significantly reduced susceptibility to chemical hydrolysis compared with aliphatic esters [135].

An attempt to FDM 3D print small biodegradable pots from PCL with the addition of collagen hydrolysate proved to be an excellent solution, ensuring complete material degradation within 30 days. The authors tested the biodegradability of PCL/HA blend filaments against a reference material, cellulose, with significantly better results [205]. The addition of plant biomass, soybean waste, to PLA resulted in the printing of pots suitable for planting in soil. The developed formulations were tested on plants (tomato seedlings), but the degree of biodegradation of the material in the soil was not tested [213]. 

**Table 6:** Biodegradation of 3D-printed polymeric materials during composting.

Printed Material	AM Method	T (°C)	Time (Days)	Compost Type	Indicators	Biodegradation Test Type	Biodegradation Rate	References
PLA, PHB, PLA/PHB	FDM	58	50	Thermophilic microorganisms	CO_2_ release	Laboratory scale ASTM D5338-15 [214], ISO 14852 [215]	PHB—86.4% PLA50/PHB50—85% PLA—21.7%	Choe et al. [201]
PLA, PLA/PHA	FDM	58	84	From a sorting and composting plant	CO_2_ release	Laboratory scale ASTM D6400 [216] PN-EN 14806:2010 [217]	PLA 21% PLA/PHA 30%	Rydz et al. [212]
PLA, PLA/PHA	FDM	60 61	21 21–84	BIODEGMA system static composting open-air pile, industrial system	Macroscopic visual evaluation	Industrial scale, sorting and composting plant, Zabrze, Poland	n.a.	Rydz et al. [212]
PCL/collagen hydrolysate	FDM	58	30	Olive mill waste (83 wt.% pomace and 6 wt.% leaves and twigs), waste wool (6 wt.%), wheat straw (3 wt.%), and chicken manure (2 wt.%)	Macroscopic visual evaluation	Composting pile (1 m^3^) UNI EN ISO 14045 [218]	Complete disintegration in 30 days	Seggiani et al. [219]

n.a.—not applicable.

### 3.5. Ecotoxicity of Composite Degradation Products

Biodegradable polymers can cause the accumulation of decomposition products in the environment. Complete mineralization of samples by microorganisms results in the release of water, carbon dioxide, or methane and is an environmentally friendly solution. However, incomplete degradation leads to the accumulation of oligomers, monomers, or other decomposition product forms in the environment, which affects soil-living organisms. Therefore, an essential complementary element of biodegradability research should be the study of the ecotoxicity of polymer composite decomposition products.

There are no clearly defined standards for biodegradable polymers to assess their effects on aquatic and terrestrial organisms. The European standard EN 13432 [220] for assessing compostability supplements biodegradability tests with tests on plants [5]. To estimate the impact of polymer degradation products, it is worth using screening tests that consider toxicity standards for aquatic invertebrates (daphnia) and plant phytotoxicity tests. Reports from scientists studying the toxicity of bioplastics indicate that while biopolymers (PLA and PHA) are harmless to the larvae of the sea urchin *Paracentrotus lividus*, additives such as plasticizers may pose a threat in this area [221]. Available literature on biodegradation of 3D-printed composites does not provide any information on this topic and the set of additives used to produce prints is very wide.

## 4. Conclusions and Future Perspectives

The continued development of AM technology and functional polymers is leading to a positive transformation of the manufacturing industry, thereby increasing the benefits to our society. Despite these benefits, AM technology still has to overcome several limitations, including a limited choice of printing materials due to printing requirements such as rheology, melting point, and other physical properties. According to the literature presented, 3D printing technology is leading to personalization with the ability to subdivide down to the nanoscale. As a result, the application space is expanding with new opportunities to produce high-performance products with optimized structure and function on a large scale. The ability to engineer the chemical and physical properties of polymers at an early stage offers the opportunity to activate shape-shifting and control the movement of printed products. A multi-material, multi-scale manufacturing technique is needed to simultaneously control the composition and proportions of materials and functions, as well as the internal architecture at the micro- and nanoscale. Objects created for biological, electronic, and robotic applications typically require multiple materials at different scales to perform a series of complex motions or numerous components for specific reactions. Composite materials are used in various applications and it is often necessary that they are completely degraded in the final stage without leaving residues in the environment. 

Studies related to the biodegradability analysis of composites should provide clear information on the degree of degradation and the conditions under which the process occurs. To meet these requirements, it is necessary to implement a variety of measures, some of which are outlined here. Further studies are needed to evaluate the effects of printing conditions and biodegradable polymer additives on ink degradation under different conditions. Individual studies in this area confirm the relationships between factors. 

Standardization of biodegradability assessment methods appears to be a necessary step, as there is currently no universal method by which a product can be considered fully degradable and safe for the environment. Degradation tests involve the analysis of various indicators, which do not always reflect the actual state of the sample. Incorporating several types of tests simultaneously provides a better chance of effectively assessing the degree of degradation. It is crucial to verify the effectiveness of biodegradation under natural conditions, which provides a complete overview of the fate of the product in real ecosystems. Aquatic biodegradation tests are typically conducted in controlled laboratories, which have limited ability to replicate natural water conditions. To fully assess the biodegradation potential in aquatic environments, tests should mimic different freshwater and marine environments, including different climatic zones, to reflect real-world scenarios. It is also necessary to compare the biodegradation rate of the same samples in different environments (water, soil, and compost). In this way, the most favorable conditions can be offered. The evaluation of the environmental impact of biodegradable products is crucial, and the use of standardized ecotoxicity tests is essential to study incomplete biodegradation. 

It is also essential to selectively collect biodegradable materials that can be decomposed under certain conditions to ensure complete degradation. However, this step requires awareness campaigns, which would be a good step toward the conscious choice of environmentally friendly products.

## Figures and Tables

**Table 1:** Functional additive-reinforced polymer composites produced using AM technology.

3D Printing Method	Type of Materials	References
FDM	Copper/ABS, iron/ABS	Nikzad et al., (2011) [15] Hwang et al., (2015) [17]
	Wood/PLA	Ayrilmis et al., (2019) [18]
	Al and Al_2_O_3_/Nylon-6	Boparai et al., (2015) [19]
	BaTiO_3_/ABS	Castles et al., (2016) [20]
	Tungsten/PC	Shemelya et al.(2015) [21]
	TPE/ABS	Perez ART et al., (2014) [22]
	Short glass fiber/ABS	Zhong et al., (2001) [23]
	Short carbon fiber/AB	Tekinalp et al., (2014) [24]
	Glass fiber/PP	Ning et al., (2015) [25]
	Microspheres/polywax	Carneiro et al., (2015) [26]
	VGCFs/ABS	Wang et al., (2016) [27]
	SWNTs/ABS	Shofner et al., (2003) [28]
	Recycled wood fibers/PLA and PHA matrix carbon fibers/PLA, natural jute fibers/PLA	Le Duigou et al., (2016) [29] Matsuzaki et al., (2016) [30]
	Continuous carbon fiber/PLA	Li et al., (2016) [31]
	Carbon fibers/ABS	Nakagawa et al., (2017) [32]
	Continuous carbon fiber/ABS/resin	Zhong et al., (2001) [33]
	Montmorillonite/ABS	Weng et al., (2016) [34]
	Graphene/ABS	Wei et al., (2015) [35]
	poly epsilon-caprolactone (PCL)	Zein et al., (2002) [36]
		Martin et al. [37]
DLP	Alumina/UV-sensitive resin	Kokkinis et al., (2015) [38]
Direct writing with magnetic assistance	Alumina/polyurethane acrylate	Compton et al., (2014) [39]
Direct write	Short carbon fiber/SiC whisker/epoxy	Van Der Klift et al., (2016) [40]
DDM	Continuous carbon fiber/nylon	Yan et al., (2011) [41]
SLS	PA12+nanokrzemionka	Chung et al., (2006) [42]
	Glass bead/Nylon-11	Goodridge et al., (2011) [43]
	Carbon nanofibre-polyamide-12	Lin et al., (2015) [44]
	Graphene oxide/photopolymer	Yugang et al., (2011) [45]
	TiO_2_/epoxy acrylate	Kim et al., (2014) [46]
	BaTiO_3_/PEGDA	Zhang et al., (2018) [47]
	CNT/acrylic ester	Athreya et al., (2010) [48]
	Carbon black/nylon-12	Zheng et al., (2006) [49]
	Al_2_O_3_/polystyrene	Kim et al., (2013) [50]
	Cont. carbon-TiO_2_/nylon-12 and graphite/nylon-12	Lin et al., (2014) [51]
	Graphene oxide (GO)/iron (Fe)	Kurimoto et al., (2015) [52]
SLA	Al_2_O_3_/UV-cured resin	Kalsoom et al., (2016) [53]
	Diamond microparticle/acrylate resins	Hector et al., (2006) [54]
	CNT/epoxy	Zanchetta et al., (2016) [55]
	Silicon oxycarbide (SiOC) (PDCs)	Suwanprateeb et al., (2006) [56]
	Starch-based polymer powders (starch/cellulose fiber/sucrose sugar/maltodextrin)	Guo et al., (2015) [57]
Solvent-cast direct writing	CNT/PLA	Krivec et al., (2017) [58]
Inkjet printing	Ag/photopolymer	Shao et al., (2016) [59]
	CSi-Mg/TCP	Wu et al., (2011) [60]
	MBG powder (Si/Ca/P)/PVA	Bergmann et al., (2010) [61]
	Bioactive glass/β-TCP	Lam et al., (2002) [62]
PLP (3DP)	Starch-based polymer powders (cornstarch/dextran/gelatin)	Zhou et al., (2018) [63]
	HA/CaSO_4_ powder with PCL infiltration	Vaezi et al., (2011) [64]
	Powder (ZP102)/binder (Zb56)	Glasschroeder et al., (2015) [65]
	PMMA/screw nuts and PMMA/carbon fibers	Hui et al., (2018) [66]
LS	nHA/PA12	Schwentenwein et al., (2015) [67]
LCM	Alumina ceramics	Nikzad et al., (2011) [15] Hwang et al., (2015) [17]

**Table 2:** Overview of 3D-printed multifunctional composites.

Property Type	3D Printing Method	Additional Material	Intentions	Application	Reference
Thermal properties	FDM	Boron nitride	Dispersion quality	Heat exchangers	Quill et al. [69] Liu et al. [70] Belaid et al. [71] Su et al. [72] Peng et al. [73]
	FEAM	Synthetic microdiamonds	Performance	Heat sinks	Kowalewska et al. [74] Wang et al. [75] Bogdanov et al. [76] Yaragatti et al. [77]
Conductive properties	FDM	CNT	Viscosity	Electrical conductors	Jariwala et al. [78] Ghoshal [79] Gnanasekaran et al. [80] Yang et al. [81] Lage-Rivera et al. [82] Omar et al. [83]
	DIW	Graphene	Dispersion quality	Self-sensing composites	Marconi et al. [84] Martinez et al. [85] Nassar et al. [86] Tandel et al. [87] Haney et al. [88] Shao et al. [89]
	DLP	GO	Percolation threshold		Lin et al. [44] Tilve-Martinez et al. [90] Ajiteru et al. [91] Zheng et al. [92]
Embedded circuitry	FDM	Silver particles	Sintering temperature	Electrical devices	Kidalov et al. [93] Flores et al. [94] Bressan et al. [95] Calamak et al. [96]
	Inkjet	Copper particles	Multiprocessing	Photovoltaics	Raut et al. [97] Li et al. [98] Kim [99] Beedasy et al. [100] Zareei et al. [101]
Magnetic properties	FDM	Iron particles	Viscosity	Magnetic sensors	Zhang et al. [102] Afshari et al. [103]
		MnAlC particles	Dispersion quality	EMIf shields	Bekas et al. [7,104] Ehrmann et al. [105] Wang et al. [106] Vucemilovic et al. [107]
Sensing	FDM	Silver particles	Repeatability	Damage detection	Li et al. [108] Khosravani et al. [109] Omar et al. [83] Li et al. [110] Nyabadza et al. [111] Liu et al. [112]
	TEAM	Carbon black	Accuracy	Structural health	Monteiro et al. [113] Zhai et al. [114] Xia et al. [115]
	Inkjet	CNT	Performance	Monitoring	Alshammari et al. [116] Kuzubasoglu et al. [117] Yuan et al. [118]
Self-healing	FDM	Re-mendable polymer	Capsule development	Autonomous structures	Platonova et al. [119] Almutairi et al. [120] Snyder et al. [121]
	DIW		Vascule development		Qamar et al. [122] Shields et al. [123] Hansen [124]

**Table 3:** Thermoplastics filled with plant-based materials and manufactured using AM technology.

3D Printing Method	Fibers	References
FDM	Cellulose	Dong et al. [143] Tekinalp et al. [144]
	Microcellulose and PEG 6000	Wang et al. [145]
	Poplar/glycerol/tributyl citrate	Xie et al. [146]
	Galactomannan(GM) from spruce thermomechanical pulp	Xu et al. [147]
	Beechwood	Kariz et al. [148]
	Microcrystalline cellulose	Murphy et al. [149]
	Native and partially delignified fibrillated beechwood	Winter et al. [150]
	Rice husk flour, pine wood flour	Le Guen et al. [151]
	TEMPO-oxidized bacterial cellulose	Chen et al. [152]
	Poplar wood flour + tributyl citrate	Lin et al. [153]
	Pulp, wood, or kraft lignin < 300 mesh	Liu et al. [154]
	Pine kraft lignin, beech organosolv lignin, or beech lignosulfonate	Mimini et al. [155]
	PBAT + hemp + EGMA (Lotader AX8900) + lubricant, antioxidant and anti-hydrolysis agent	Xiao et al. [156]
	Poplar	Zhao et al. [157]
	Alkaline spruce lignin	Tanase-Opedal et al. [142]
	Sugarcane bagasse and cellulose extracted from bagasse	Liu et al. [158]
	Ball-milled poplar	Bhagia et al. [159]
	Acetylated tannin	Liao et al. [140]
	Recycled—PLA + microcrystalline cellulose and Joncryl chain extender	Cisneros-López et al. [160]
	PHA + wood (commercial filament)	Le Duigou et al. [29]
	Cellulose (silanized) + PEG6000	Wang et al. [145]
	Poplar + glycerol + tributyl citrate	Xie et al. [146]
	Galactomannan	Pranovich et al. [147]
	Wood	Dong et al. [161]
	Beechwood	Kariz et al. [148]
	1% native and partially delignified fibrillated beechwood	Winter et al. [150]
	PLA/PHA + pinewood	Guessasma et al. [162]
	Rice husk flour or pine wood flour	Le Guen et al. [151]
	Pulp, wood, or lignin silanized with KH550 silicone oil	Liu et al. [158]
	Pine kraft lignin, beech organosolv lignin, or beech lignosulfonate	Mimini et al. [155]
	PLA + PBAT (2003F) + hemp + EGMA (Lotader AX8900 + lubricant, antioxidant and anti-hydrolysis agent	Xiao et al. [156]
	Alkali spruce lignin	Tanase-Opedal et al. [142]
	Sugarcane bagasse or cellulose extracted from bagasse	Liu et al. [158]
	Acetylated mimosa tannins	Liao et al. [140]
	Agave fibers	Figueroa et al. [163]
	Kenaf	Shahar et al. [164] Jamadi et al. [165] Aumnate et al. [166]
	Kenaf cellulose	Liu et al. [158]
	Astragalus	Yu et al. [167]
	Natural rubber	Fekete et al. [168]
	Bamboo and flax fiber	Depuydt et al. [169]
	Wood-flour-filled fiber	Tao et al. [47]
	Soy hulls and soy protein	Dey et al. [170]
	Hemicellulose composite	Shi et al. [171]
	Bamboo compounded fiber	Long et al. [172]
	Compound of wood with plastic	Kariz et al. [148]
	Compound of straw with plastic	Yu et al. [173]
	Compound of cellulose	Ambone et al. [174]
	Lignin with plastic	Ryu et al. [175]
	Wood plastic wire	Yang et al. [176]
	Wood plastic composite	Liu et al. [154], Rahim et al. [177] Tascioglu et al. [178], Fico et al. [179], Cano-Vicent et al. [180], Baechle-Clayton et al. [181]
	Bamboo wood	Muller et al. [182]
	Straw	Yu et al. [183]
	Wheat	Zheng et al. [184]
	Corn	Paggi et al. [68]
	Galactoglucomannan	Xu et al. [147]
	Paper	Travitzky et al. [185]
	Wood chips	Rosenthal et al. [186]
SLA	Epoxy acrylate soybean oil (AESO)	Rosa et al. [187]
	Lignin-based photosensitive resins	Sutton et al. [188]
SLS	Wood plastic pellets	Zhang et al. [189]

## Data Availability

Not applicable.

## Abbreviations

FDM	Fused deposition modeling
PBAT	Polybutylene adipate terephthalate
PBS	Phosphate-buffered saline
PBS	Polybutylene succinate
PCL	Polycaprolactone
PE-g-MAH	Maleic anhydride grafted polyethylene
PHA	Polyhydroxyalkanoate
PLA	Polylactic acid
PLGA	Poly lactic-co-glycolic acid
PP	Polypropylene
HAp	Hydroxyapatite
